# Supplementary figures and images for: Predation experiments with 3D-printed lizard models yield limited responses in pheasants
Source: PeerJ. 2025 Nov 3;13:e20103. doi: 10.7717/peerj.20103 (PMC12591047; doi:10.7717/peerj.20103)

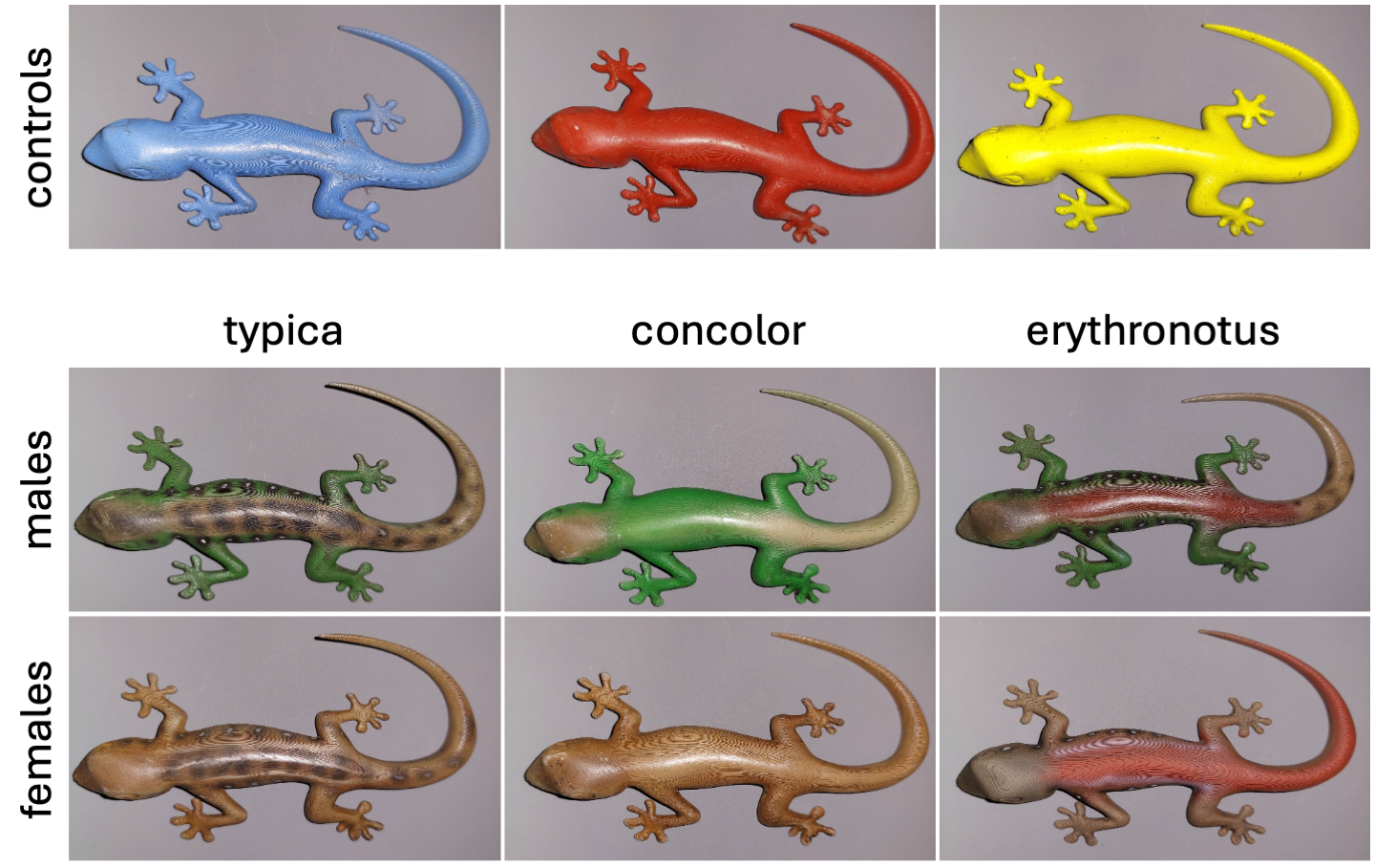

Supplement: Supplemental Information 5 [file peerj-13-20103-s005.png]

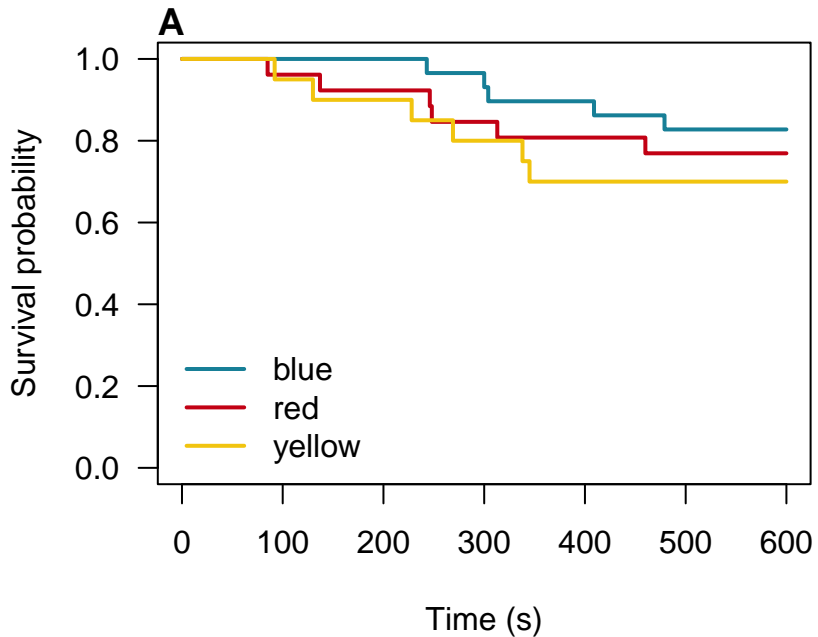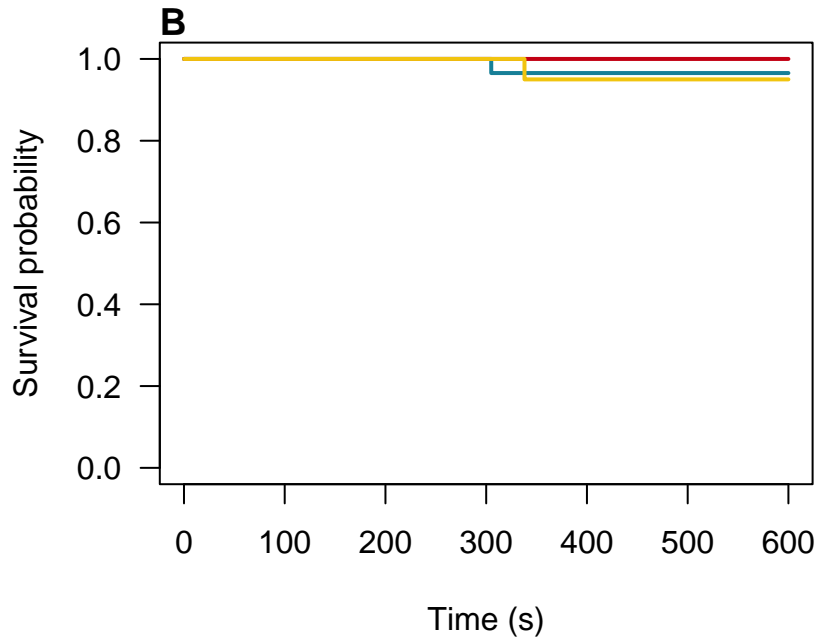

Supplement: Supplemental Information 6 [file peerj-13-20103-s006.pdf]

A

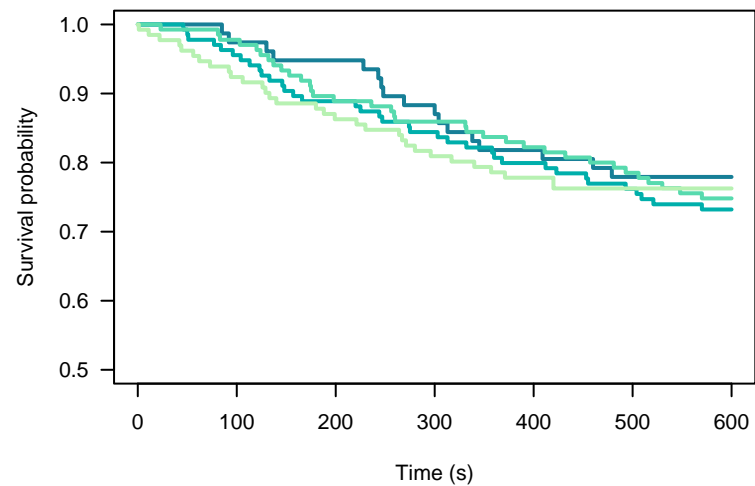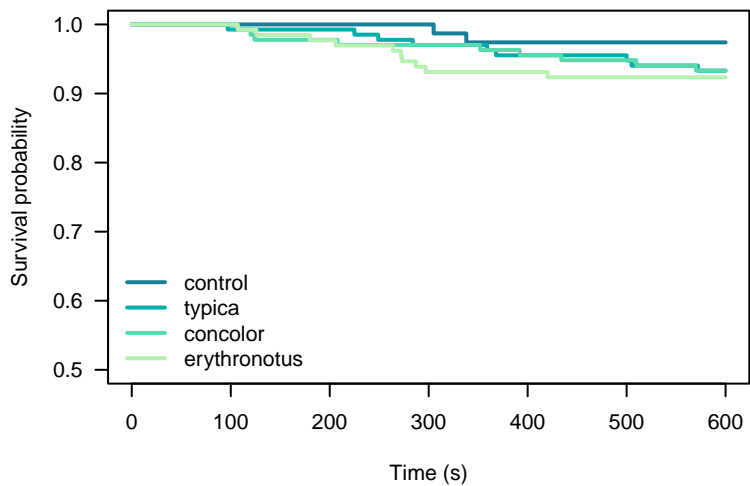

B

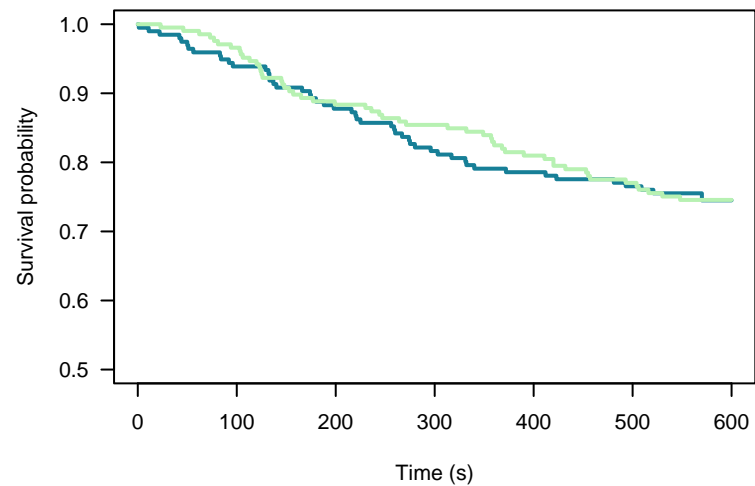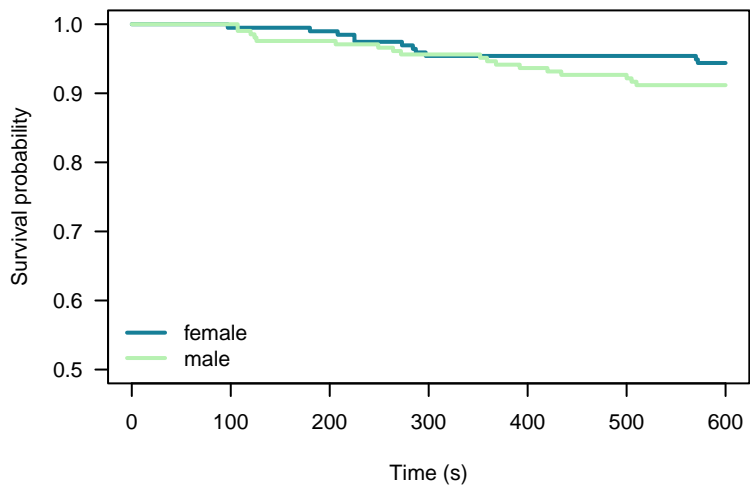

C

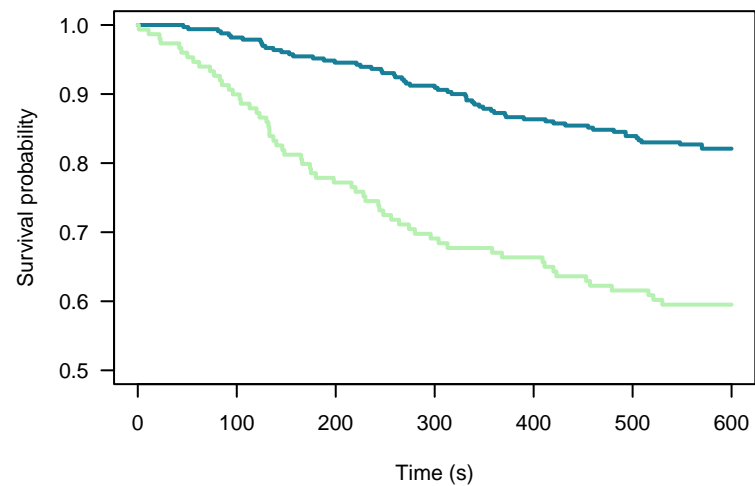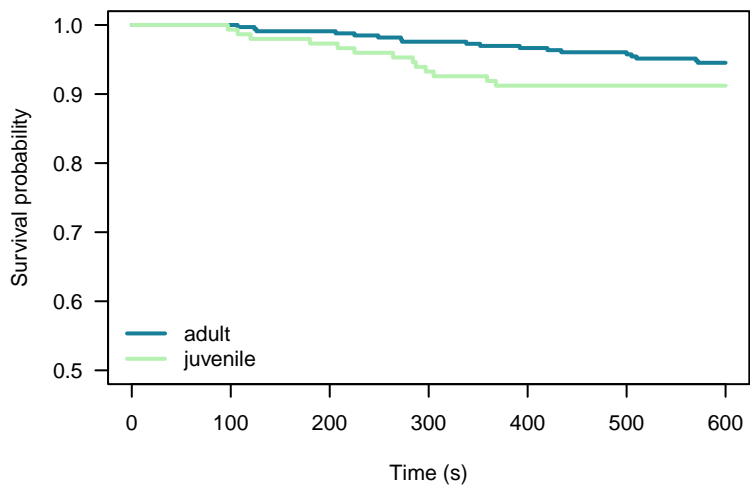

D

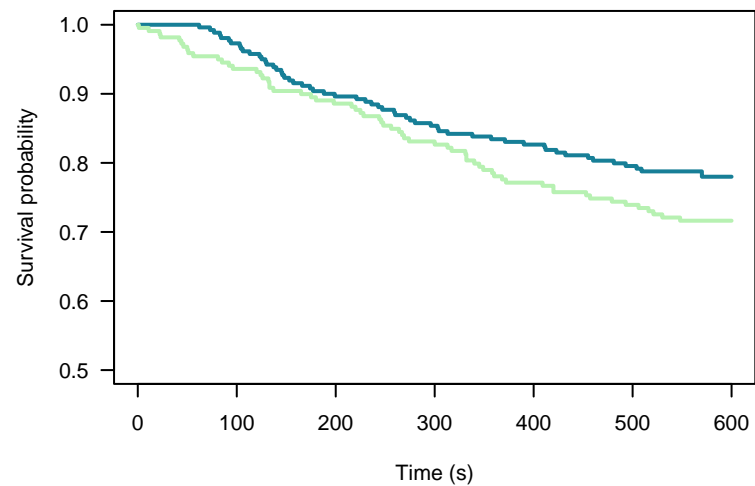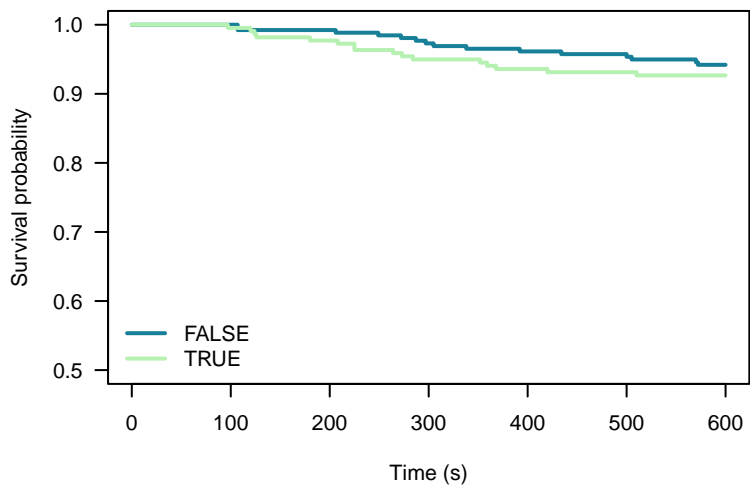

Supplement: Supplemental Information 7 [file peerj-13-20103-s007.pdf]
